# Structural requirements for strengthening the Family Health Strategy and care coordination

**DOI:** 10.1590/0102-311XEN027926

**Published:** 2026-06-22

**Authors:** Natalia Houghton, Ernesto Bascolo

**Affiliations:** 1 Pan American Health Organization, Washington DC, U.S.A.

The paper by Giovanella et al. ^1^ provides a valuable institutional analysis of the 30-year trajectory of the Family Health Strategy (FHS), documenting achievements in expanding coverage, promoting equitable access and improving health outcomes, while also acknowledging implementation gaps and periods of uncertainty. The overview of key milestones and setbacks, from the introduction of the Family Health Program in 1994 to contemporary challenges, provides a historical context that is fundamental to understand the Brazilian Unified National Health System (SUS, acronym in Portuguese) and the country’s approach to primary health care (PHC).

In the Americas, Brazil is considered a benchmark for PHC reform, due to both the SUS and the expansion of the FHS ^2^, which makes the lessons learned all the more relevant for other countries in the region. The FHS is also one of the most extensive community-based PHC programs in the world, and has been shown to have a positive impact on mortality, access to care and health inequalities ^3^.

At the same time, the article ^1^ identifies persistent structural barrier: workforce instability; underfunding; difficulties hampering the effective coordination of care; and structural issues related to equity. In particular, the authors highlight that barriers to fulfilling PHC’s coordinating function persist, despite three decades of FHS development. This challenge warrants further examination because perhaps the most significant gap between what the FHS model proposes and what is implemented in practice is in care coordination.

This debate seeks to delve deeper into these analytical questions, exploring what institutional arrangements could guarantee the sustainability of the FHS founding principles.

## Care coordination

Recent evidence supports the arguments presented in the paper and is consistent with the regional assessment of service fragmentation: challenges in ensuring smooth patient flow between primary and specialized care persist within the SUS. Timely access to complex services remains a point of tension in many networks ^4^. These fragmentation issues are not unique to Brazil, having prompted the Pan American Health Organization (PAHO), the World Bank and the Inter-American Development Bank (IDB) to develop an updated conceptual and operational framework for Integrated Health Service Delivery Networks (IHSDN), which represent operational expressions of PHC-based health systems in the Americas ^5^.

Qualitative study ^6^ involving Brazilian specialists have documented that the referral process can be affected by flaws in communication, lack of standardized protocols and limited information sharing across levels of care. More recently, analyses focusing on the FHS indicate that the challenge of consolidating “network-based care” remains, despite advances in policy and coverage ^7^. These findings are consistent with the updated IHSDN framework, which highlights that care coordination and the bidirectional flow of information are essential attributes of integrated networks, alongside territorial management and multilevel governance ^5^.

Fragmentation leads to inefficiencies and suboptimal patient experiences. For example, the paper ^1^ highlights that a significant proportion of diagnostic tests requested in primary care services are repeated in specialized services, largely due to problems with the transfer of clinical information, with cost and diagnostic turnaround time implications. The paper ^1^ also highlights that while the adoption of electronic health records has expanded, interoperability remains limited, which is an issue that the IHSDN framework addresses, highlighting that digital transformation is an enabling condition for strengthening continuity of care ^5^.

In this sense, key questions should focus on identifying which structural conditions enable progress towards network maturation, with international and regional experiences pointing to relevant dimensions. First, decentralized governance and defining responsibilities across different levels of government require clear mechanisms for intergovernmental coordination of network management, a central aspect of the IHSDN approach ^5^. In the case of Brazil, where PHC is the responsibility of municipal governments and many specialized services depend on state health authorities, strengthening shared management agreements and joint planning forums is particularly important.

Second, financing mechanisms can progressively evolve from a focus on service volume toward arrangements that explicitly take into account outcomes related to coordination and continuity of care across levels. This is consistent with the PAHO recommendation that the region should move toward payment models that promote and support functional coordination across networks, while taking into account existing budget constraints ^5^.

Third, health information system organization and utilization remain a critical component. The COVID-19 pandemic highlighted the importance digital transformation, also spurring innovations that offer potential solutions. For example, the Regula Mais Brasil Project, aimed at improving the scheduling of patient access to health services in Recife (Pernambuco State), used teleconsultations between PHC teams and specialists to assess referrals and manage demand, reducing wait times and improving the use of installed capacity ^7^. This type of initiative is consistent with the emphasis placed on telehealth by the IHSDN framework as a tool to support the coordination of clinical care and territorial organization of services ^5^.

Finally, the paper ^1^ highlights the growing use of e-SUS Território, an app that helps community health workers to collect and update resident and household data. Digital tools have demonstrated the ability to improve data collection, team coordination and population monitoring, constituting an important asset for integrated network management. However, digitalization can also lead to increased workloads and new forms of work-related stress if not accompanied by adequate workforce protection and support frameworks. From the IHSDN perspective, the challenge is to ensure that digital transformation translates into in better outcomes for the population and better working conditions for health teams, reinforcing the role of primary care and the FHS as cornerstones of care coordination.

## Toward institutional consolidation

The study ^1^ highlights that the FHS has faced significant policy changes, including a shift in the focus of financing mechanisms and changes in emphasis between territorial and individual care. These shifts raise a key question: Which institutional arrangements ensure that the underlying principles of the FHS - equity, territorialization, community-centered approach and care coordination - are upheld in the face of a changing fiscal environment and political priorities?

A comparative analysis of enduring policies suggests that the foundational elements of the system are clearly protected by legislation, funding mechanisms based on service needs and governance structures that safeguard core functions against short-term fluctuations. The challenge for Brazil’s health system is to move toward structural consolidation: arrangements that ensure the foundational principles of the FHS translate into effective care coordination, network integration and the reduction of geographical and social inequities.

This discussion calls for a more in-depth examination of these institutional requirements, placing care coordination at the forefront of the agenda for strengthening the FHS.

## Data Availability

Not applicable.
