# Supplementary material for: Structural requirements for strengthening the Family Health Strategy and care coordination
Source: Cad Saude Publica. 2026 Jun 22;42:e00027926. doi: 10.1590/0102-311XEN027926 (PMC13286395; doi:10.1590/0102-311XEN027926)
Supplement: Translation

# Translation: Requisitos estructurales para la consolidación de la Estrategia de Salud Familiar y coordinación del cuidado

**DOI:** 10.1590/0102-311XES027926

**Authors:** Natalia Houghton, Ernesto Bascolo

**Affiliations:** 1 Pan American Health Organization, Washington DC, U.S.A.

El artículo de Giovanella et al. ^1^ ofrece un valioso análisis institucional de 30 años de la Estrategia de Salud Familiar (ESF), documentando logros en la expansión de cobertura, equidad en el acceso y mejoras en salud, al tiempo que reconoce brechas en la implementación y períodos de incertidumbre. La revisión de hitos clave, desde el Programa Salud de la Familia de 1994 hasta los desafíos contemporáneos, proporciona un contexto histórico fundamental para comprender el sistema de salud brasileño y su enfoque de atención primaria de salud (APS).

En las Américas, Brasil es considerado un caso emblemático de reforma orientada a la APS, tanto por la creación del Sistema Único de Salud (SUS) como por la expansión de la ESF ^2^, lo que hace que sus lecciones sean particularmente relevantes para el resto de los países de la región de las Américas. La ESF constituye, además, uno de los programas de APS comunitaria más extensos del mundo, con efectos demostrados en mortalidad, acceso y reducción de desigualdades ^3^.

Al mismo tiempo, el artículo ^1^ identifica barreras estructurales persistentes: inestabilidad de la fuerza de trabajo, insuficiencia del financiamiento público, dificultades en la coordinación efectiva del cuidado y problemas estructurales en términos de equidad. Especialmente, señala que persisten barreras para que la APS ejerza plenamente su función de coordinación a pesar de tres décadas de desarrollo de la ESF. Este desafío merece un examen más profundo, ya que la coordinación de la atención representa, quizá, la brecha más importante entre el modelo propuesto de la ESF y su implementación.

Este artículo de debate busca profundizar en dichas preguntas analíticas, explorando qué arreglos institucionales podrían garantizar la sostenibilidad de los principios fundacionales de la ESF.

## Coordinación de la atención 

La evidencia reciente ilustra los planteamientos del ensayo y es coherente con el diagnóstico regional sobre fragmentación de los servicios: en el SUS persisten desafíos para la articulación fluida entre el primer nivel de atención y la atención especializada. El acceso oportuno a servicios de mayor complejidad continúa siendo un punto de tensión en muchas redes ^4^. Estos problemas de fragmentación no son exclusivos de Brasil; han motivado como respuesta el desarrollo, por parte de la Organización Panamericana de la Salud (OPS), el Banco Mundial y el Banco Interamericano de Desarrollo (BID), del marco conceptual y operativo actualizado sobre Redes Integradas de Servicios de Salud (RISS) como expresión operativa de los sistemas de salud basados en la APS en las Américas ^5^.

Estudio cualitativo ^6^ con especialistas brasileños ha documentado que el proceso de derivación puede verse afectado por fallas de comunicación, ausencia de protocolos estandarizados y transferencia limitada de información entre niveles. Más recientemente, análisis centrados en la ESF, señalan que la consolidación de la “atención en red” sigue siendo un reto pendiente, a pesar de los avances normativos y de cobertura ^7^. Estos hallazgos dialogan con el marco actualizado de RISS, que destaca la coordinación de la atención y el flujo bidireccional de información como atributos esenciales de redes integradas, junto con la gestión territorial y la gobernanza multinivel ^5^.

La fragmentación se traduce en ineficiencias y experiencias subóptimas para los usuarios. Por ejemplo, el artículo ^1^ discute que una proporción importante de exámenes diagnósticos solicitados en la APS se repite en el nivel especializado, en buena medida por problemas en la transferencia de información clínica, con implicaciones en costos y tiempos de diagnóstico ^1^. El texto también señala que la adopción de historias clínicas electrónicas se ha expandido, aunque persisten limitaciones de interoperabilidad ^1^, un tema que el marco de RISS aborda al destacar la transformación digital como palanca para mejorar la continuidad de la atención ^5^.

En este contexto, las preguntas clave se orientan a identificar qué condiciones estructurales permiten avanzar hacia redes más maduras. La experiencia internacional y regional sugiere dimensiones relevantes. En primer lugar, la gobernanza descentralizada y la distribución de competencias entre niveles de gobierno plantean la necesidad de mecanismos explícitos de coordinación intergubernamental para la gestión de redes, un aspecto central del enfoque de RISS ^5^. En el caso brasileño, donde la APS es responsabilidad municipal y muchos servicios especializados dependen de instancias estatales, fortalecer acuerdos de gestión compartida y espacios de planificación conjunta resulta particularmente pertinente.

En segundo lugar, los mecanismos de financiamiento pueden evolucionar progresivamente desde esquemas centrados en volumen de servicios hacia arreglos que incorporen explícitamente resultados de coordinación y continuidad del cuidado entre niveles. Esto es consistente con las recomendaciones de la OPS sobre avanzar hacia modelos de pago que promuevan y apoyen la integración funcional de las redes, sin desconocer las restricciones fiscales existentes ^5^.

En tercer lugar, la organización y el uso de los sistemas de información en salud siguen siendo un componente crítico. La pandemia de COVID-19 puso en evidencia la importancia de estas transformaciones, y también generó innovaciones que ofrecen pistas de solución. El proyecto Regula Mais Brasil en Recife (Estado de Pernambuco), por ejemplo, utilizó teleconsultas entre equipos de APS y especialistas para cualificar derivaciones y gestionar la demanda, reduciendo tiempos de espera y mejorando el uso de la capacidad instalada ^7^. Iniciativas de este tipo son coherentes con el énfasis que el marco regional de RISS otorga a la telesalud como herramienta para apoyar la coordinación clínica y la organización territorial de los servicios ^5^.

Finalmente, el artículo ^1^ destaca la expansión de la utilizaciónde e-SUS Território entre agentes comunitarios de salud. Las herramientas digitales han demostrado capacidad para mejorar el registro de datos, la coordinación de equipos y el monitoreo poblacional, y constituyen un activo importante para la gestión de redes integradas. Al mismo tiempo, la digitalización puede ir acompañada de intensificación del trabajo y nuevas formas de presión laboral si no se acompaña de marcos adecuados de protección y apoyo a la fuerza laboral. Desde la perspectiva de las RISS, el desafío es asegurar que la transformación digital se traduzca en mejores resultados para la población y en mejores condiciones de trabajo para los equipos de salud, reforzando los principios de la atención primaria y de la ESF como ordenadoras de la atención.

## Hacia la consolidación institucional

El artículo ^1^ documenta que la ESF enfrentó cambios importantes en la política, incluyendo cambios en los mecanismos de financiamiento y fluctuaciones en el énfasis territorial versus individual. Estas transiciones plantean una pregunta clave: ¿qué arreglos institucionales aseguran que los principios fundacionales de la ESF -equidad, territorialización, enfoque comunitario y coordinación del cuidado- persistan frente a diferentes climas fiscales y prioridades de política?

El análisis comparativo de políticas duraderas sugiere que los elementos fundacionales del sistema se benefician de protecciones legislativas claras, mecanismos financieros dedicados indexados a la necesidad del servicio y estructuras de gobernanza que protejan funciones centrales de variaciones coyunturales. El desafío para el sistema de salud brasileño es avanzar hacia una consolidación estructural: arreglos que garanticen que los principios fundacionales de la ESF se traduzcan de manera sostenible en coordinación efectiva del cuidado, integración de redes y reducción de inequidades territoriales y sociales.

Este debate invita a un examen más profundo de estos requisitos institucionales, situando la coordinación del cuidado en el centro de la agenda de fortalecimiento de la ESF.

## Data Availability

No aplicable.
